# Eating disorder risk in adolescent and adult female athletes: the role of body satisfaction, sport type, BMI, level of competition, and training background

**DOI:** 10.1186/s13102-023-00683-7

**Published:** 2023-07-25

**Authors:** Joanna Borowiec, Adrianna Banio-Krajnik, Ewa Malchrowicz-Mośko, Adam Kantanista

**Affiliations:** 1 Department of Physical Education and Lifelong Sports, Poznan University of Physical Education, Królowej Jadwigi 27/39, Poznań, 61–871 Poland; 2 Institute of Physical Culture Sciences, Faculty of Physical Education and Health, University of Szczecin, 70-453, Szczecin, Al. Papieża Jana Pawła II 22a, Szczecin, Poland; 3 Department of Sport Tourism, Poznan University of Physical Education, Królowej Jadwigi 27/39, Poznań, 61–871 Poland

**Keywords:** Eating disorders, Body image, Young athletes, Aesthetic, Non-aesthetic, Ball sports

## Abstract

**Background:**

Eating disorders negatively influence athletes’ health and performance. To achieve a high level of performance and conform to cultural expectations regarding an athletic body type, female athletes often restrict their diets, which can lead to eating disorders. In addition to factors related to the sports environment, adolescent athletes are subject to changes caused by the maturation process. Therefore, the same factors may have different effects on eating disorder risk among adolescent and adult athletes. This study examined the relationship between eating disorder risk, specific aspects of the sports environment (sport type, level of competition [national and international], and training background), and individual aspects (body satisfaction and body mass index) in two groups of athletes: adolescents and adults.

**Methods:**

The sample included 241 highly trained female athletes aged 12–30 years *(M =* 20.68, *SD =* 4.45) recruited from different sports clubs in Poland. The subgroup of adolescents consisted of 82 athletes, while the number of adult athletes was 159. The Eating Attitudes Test questionnaire was used to assess the eating disorder risk among the athletes. Body satisfaction was measured using the Feelings and Attitudes Toward Body Scale incorporated into the Body Investment Scale.

**Results:**

Eating disorder risk was prevalent among 14.6% of the adolescent and 6.9% of the adult athletes. Significant associations between eating disorder risk and the studied variables were noted only among adolescent athletes. Univariate logistic regression analysis revealed that the occurrence of eating disorder risk was associated with participation in lean non-aesthetic sports (OR = 11.50, 95% CI: 3.58–37.09). Moreover, eating disorder risk was associated with athletes’ lower body satisfaction (OR = 0.80, 95% CI: 0.70–0.92). Body mass index was not included in the final regression model.

**Conclusions:**

The study indicated that eating disorder risk in adolescent female athletes was related to sport type and body satisfaction. The findings showed that, in adolescent athletes, eating disorder risk was the most associated with practicing lean non-aesthetic sports. Coaches and athletes should be aware that eating disorder risk increases among individuals with a lower body image.

## Background

Eating disorders are prevalent among athletes, especially on the competitive and elite levels [1–4]. Restrictive dieting often turns into chronic dieting, then into disordered eating, which refers to various abnormal eating behaviors, such as restrictive eating; fasting; frequently skipping meals; taking diet pills, laxatives, diuretics, or enemas; overeating; and purging [5, 6]. At the end of this continuum are clinical eating disorders, such as bulimia nervosa, anorexia nervosa, binge-eating disorder, and other specified feeding and eating disorders [7].

Statistics concerning the prevalence of eating disorders among athletes are alarming and show that this issue requires extensive research to identify its determinants. For example, Flatt et al. [8] examined 3509 male and female competitive athletes and reported that 74% of them reported binge eating, 26% reported vomiting, and 50% reported fasting. In a recent study by Ravi et al. [5] among 846 female athletes representing 67 different sports, 25% reported restrictive eating and 18% reported eating disorders. Likewise, Borja et al. [6] found that 473 (47.3%) out of 1000 female athletes scored high on eating disorder questionnaires. Eating disorders are dangerous and can sabotage sporting success and seriously damage athletes’ physical and mental health [9].

There are many specific conditions in sport that influence the emergence of eating disorders among athletes. To win a sports championship, most athletes train for many hours a day, consuming a lot of energy, which increases their daily caloric requirement [10]. Unfortunately, this energy demand is not always met by diet, because in many sports, a slim figure and a lower body weight are conducive to achieving better results [11]. It should be noted that there are also sports, such as technical, power, and ball sports [7], which promote different body ideals and are not lean body oriented. However, in some studies, disordered eating and eating disorders were also found among athletes of non-lean disciplines [7, 12]. Originally, studies investigating eating disorders among athletes focused mainly on the aesthetic and non-aesthetic division. However, this approach turned out to be insufficient and did not provide a complete picture of eating disorders. The division of sports into lean and non-lean is more adequate in eating disorders analyses. Specification may be higher when sports are further divided into aesthetic, weight-dependent, endurance, ball, power, and technical sports [7]. Mancine et al. [7] postulated the need to conduct research on various sport types to accurately identify relationships and risk factors of eating disorders.

Many eating problems in athletes start at a young age [5, 13]. Adolescents experience developmental processes that are no longer observed in adulthood. When both groups are combined, the impact of factors affecting only one of the groups may not be captured by statistical methods. Therefore, it is worthwhile to examine adolescents and adults separately.

### Eating disorders and body image

In examining athletes, researchers found significant negative relationships between eating disorders and body image [3, 4, 12]. De Oliviera et al. [14] verified that most athletes in various sports were dissatisfied with their body image. Beals and Manore [15] found that athletes with eating disorders were significantly more dissatisfied with their bodies than those without disorders. Initially, the relationship between eating disorders and body dissatisfaction was mainly reported among athletes in aesthetic sports that emphasized a slim figure, such as gymnastics, bodybuilding, cheerleading, artistic swimming, ballet, and figure skating [16–18]. In recent years, more attention has been paid to non-aesthetic sports, such as endurance sports and sports with weight categories [1, 18–20]. For example, Beals and Manore [15] found lower body satisfaction and eating disorder symptoms among 75% of participants who participated in endurance sports. Fochesato et al. [12] also reported lower body satisfaction (30% of participants) and eating disorder behaviors (16% of participants) in a cohort of female athletes in volleyball sports clubs.

Studies have highlighted athletes’ tendency to perceive their bodies in a distorted way. Even if their body weight and body mass index (BMI) were appropriate and their appearance corresponded to cultural expectations, they were often dissatisfied with their bodies [21, 22]. This is because, in some sports, exaggerated thinness is perceived as advantageous for both performance and appearance. If athletes do not meet the expectations of their sport environment, their body satisfaction may be low [3, 4]. In modeling body dissatisfaction among athletes, Krentz and Warschburger [22] underlined the importance of the discrepancy between actual and ideal body shape for sports performance. Negative body evaluations and body comparisons are an important factor in the development of eating disorders, especially in the athletic context [4]. However, there are some sports in which large, strong, and athletic body types are desired. Researchers found that female rugby players, cricketers, netballers, swimmers, and throwers felt pride in their bodies in a sport context but evaluated their bodies more negatively in relation to cultural expectations of the female body [23–25]. Although the data are inconclusive, Hausenblas and Downs [26] showed that athletes report lower body dissatisfaction than non-athletes. Athletes who are dissatisfied with their bodies and who wish to reach a desired body weight, silhouette, and physical performance subject their bodies to nutritional restrictions, which in many cases lead to eating disorders [11].

### Eating disorders and sport type

It has been extensively documented that eating disorders are highly prevalent among aesthetic sport athletes [16, 25]. In aesthetic sports, athletes are judged based on aesthetic components, such as the beauty of movements, physical attractiveness, and leanness, which is reflected in the belief that “thin is going to win” [16, p. 50 7]. However, recent studies have shown that, on the competitive level, it is not only individuals who engage in aesthetic sports that suffer from eating disorders; this problem also occurs in non-aesthetic athletes [27]. In a systematic review, Godoy-Izquierdo et al. [1] found that eating disorders were common among athletes participating in a variety of non-aesthetic sports, including individual sports, such as running, triathlon, cycling, and sports with weight categories. Barrack et al. [28] noted eating disorders among 20% of female endurance runners. Athletes who practice disciplines with weight categories are also at high risk of developing eating disorders. For example, Escobar-Molina et al. [13] analyzed the weight loss methods of 144 elite judo athletes and discovered the presence of eating disorders. This indicates that in the context of eating disorders, the division of sports into aesthetic and non-aesthetic is insufficient. Splitting sports into lean and non-lean seems to be more useful [7].

Mancine et al. [7] classified ball sports as non-lean. Ball sports are disciplines in which body weight and size do not directly determine athletic performance or success [29]; therefore, it might be assumed that eating disorders would not arise in this group of athletes. However, several studies have revealed the prevalence of eating disorders among football and volleyball players [15, 30–32]. In Noll et al.’s [33] study, among 248 ball sport athletes, 43% of adolescents and 49% of adults skipped breakfast, which was also associated with vomiting or using laxatives to lose weight. Torres-McGehee et al. [34] found that 25% of female soccer athletes suffered from binge eating. Eating disorders might occur among athletes practicing lean aesthetic and lean non-aesthetic sports and non-lean sports, including ball sports.

### Eating disorders and BMI

De Bruin’s studies [3, 16] indicated that eating disorders among athletes were related to BMI. However, the relationship between eating practices and BMI might be bidirectional; BMI affects eating behaviors and eating behaviors influence BMI [35]. There are studies which attributed a large percentage of eating disorders among athletes to BMI [36]. De Bruin et al. [16] emphasized that it was not the real BMI that influenced eating disorders among athletes but athletes’ subjective assessment of their BMI and the discrepancy between their current and desired BMI. The authors found that dieting frequency was higher when gymnasts were heavier or perceived themselves to be fat. Sundgot-Borgen and Larsen [37] revealed that athletes had a BMI within or below the optimal level; however, 31% of them continued to diet and 11% used unhealthy weight-control methods. In analyzing the determinants of eating disorders among 583 Slovenian athletes, Pustivšek et al. [38] showed that the risk group had significantly higher BMI percentiles than the group without eating disorders. However, the authors emphasized that body composition played a greater role than BMI in the development of eating disorders in athletes.

### Eating disorders, level of competition, and training background

Some studies have shown that the level of competition and training experience are related to the development of eating disorders in athletes [5, 27]. Fochesato et al. [12] found that the occurrence of eating disorders was correlated to the level at which athletes compete, with elite athletes determined to be the highest risk group. Similarly, Werner et al. [30] reported that higher-level competitive athletes show eating disorder symptoms more often than lower-level and recreational athletes. Picard [39] indicated that athletes competing at higher levels showed more signs of pathological eating and were at increased risk of developing eating disorders. This observation is in line with studies which showed that body dissatisfaction and eating disorders were prevalent among competitive female athletes [16].

When analyzing the literature to explain the relationship between training background and the occurrence of eating disorders among athletes, it can be observed that these factors have not yet been studied separately. The age of the athletes and the level of competition were most often described, and the training background was treated as their derivative. In this context, training for a sport since childhood or being an elite athlete might be related to the development of an eating disorder [40]. It is likely that athletes’ age, level of competition, and training background are closely related.

### Aim of the study

Based on the previous researches there is a need to study the risk of eating disorders also among athletes participating in lean sports that are not aesthetic and ball sports. This problem is underestimated. Thus, the aim of this study was to analyze the relationships between eating disorder risk and the type of sport (lean aesthetic, lean non-aesthetic, and ball sports), the potential vulnerability factors (such as body satisfaction, BMI) and factors related to the sport environment (level of competition and training background) among female athletes. In addition, taking into account the dynamics of developmental processes, the above relations were analyzed separately among adolescent and adult athletes. The results expand the current knowledge about eating disorder risk in each of these age groups.

## Methods

### Study population

The study population consisted of 241 highly trained Polish female adolescent (12–18 years) and adult athletes (19–30 years). The basic characteristics of the participants are presented in Table 1.

**Table 1:** Basic characteristics of the participants

Variables	All athletes N = 241	Adolescents *n* = 82	Adults *n* = 159
		*M* ± *SD*	
Age (years)	20.7 ± 4.45	15.7 ± 1.58	23.2 ± 3.10
Height (cm)	168.2 ± 10.18	164.8 ± 13.04	169.9 ± 7.83
Body weight (kg)	59.2 ± 11.49	54.0 ± 14.14	61.8 ± 8.81
Training background (years)	8.9 ± 4.60	5.8 ± 2.31	10.5 ± 4.67

*Note: M =* mean, *n =* number, *SD* = standard deviation

Among the participants, 56 (23.1%) represented lean aesthetic sports: artistic swimming (*n* = 18), gymnastics (*n* = 15), and dance (*n* = 23). Lean non-aesthetic sports were represented by 53 (21.9%) athletes participating in endurance and weight-dependent sports: athletics (*n* = 11), swimming (*n* = 14), karate (*n* = 19), and taekwondo (*n* = 8). Ball sports were represented by 133 (55%) athletes participating in soccer (*n* = 20), volleyball (*n* = 19), basketball (*n* = 21), rugby (*n* = 20), field hockey (*n* = 21), and floorball (*n* = 32). We adopted the division of sports into lean aesthetic, lean non-aesthetic, and ball sports (as non-lean sports) in line with Mancine et al. [7].

Only athletes competing at the national (32.2%) and international (67.8%) levels were included in the study. Athletes competing at other levels did not participate in the study.

### Baseline data collection

The participants were informed of the procedures and provided their written consent before their inclusion in the study. All participants took part in the study voluntarily and were informed that they could discontinue their involvement at any time. The study protocol was approved by the Local Bioethical Committee of the Karol Marcinkowski University of Medical Sciences in Poznań (No 349/20).

The authorities of 40 sports clubs, training adolescent and adult athletes at the national and international level, were asked to participate in the study. Twenty one clubs responded positively. Parents of 160 adolescent athletes were contacted with information about the study and written consent for their child’s participation in the study. Of the parents, 103 consented to their child’s participation in the study. Among adolescent athletes, out of 103 distributed questionnaires, 83 were returned (response rate was 80%). Among adult athletes researchers distributed 160 questionnaires, of which 159 were returned (response rate 99%). Finally 242 athletes (adolescents and adults) were examined in the study, of which one answer from adolescent group was not qualified for statistical analysis due to missing data in the Feelings and Attitudes Toward Body Scale questionnaire.

#### Eating disorder measurement

The Eating Attitudes Test (EAT-26) was used to assess eating disorder risk [41 EAT-26 is a self-reported questionnaire used internationally to screen for eating disorders in high school, college, and other special risk samples such as athletes. The questionnaire includes 26 questions, with 6 response options: always, very often, often, sometimes, seldom, and never. Higher EAT scores indicate increased eating pathology, with anorexia, bulimia, and other specified feeding and eating disorders all considered to be at one end of the eating pathology continuum [41]. In line with Garner et al. [42], a score of 20 or higher was used as the threshold to indicate eating disorder risk in the present study. The EAT-26 has demonstrated adequate reliability in athlete populations in previous studies [43]. The Polish adaptation of the EAT was carried out by Rogoza et al. [44]. The Cronbach’s alpha for the EAT-26 in the present study was 0.87.

#### Body satisfaction measurement

Body satisfaction was assessed using the Feelings and Attitudes Toward Body Scale incorporated in the Body Investment Scale developed by Orbach and Mikulincer [45]. The scale consists of six statements, such as “I am satisfied with my appearance” and “I feel comfortable with my body”. Participants scored each statement on a 5-point scale ranging from “absolutely disagree” to “absolutely agree” (corresponding to the point values 0–4, respectively). The total score thus ranged from 0 to 24 points; the higher the cumulative score, the more positive the athlete’s body image. We decided to use the Feelings and Attitudes Toward the Body Scale because it was adapted to the Polish population. The original scale was translated into the national language (Polish) and then translated back into English for confirmation by the Health Behavior in School-aged Children project’s International Coordinating Centre [46, 47]. This scale has been used in research on body image in adolescents and adults [48, 49]. In the present study, the scale’s internal consistency, established using the Cronbach’s alpha test, was 0.90.

#### BMI measurement

Body weight and height were self-reported by the athletes. Based on the obtained data, in the group of adult athletes, the BMI (defined as weight in kilograms divided by the square of the height in meters [kg/m^2^]) was defined. Among adolescent athletes, the age- and sex-standardized BMI (BMI z-score) was applied using the following formula:

1 $$z=\frac{{(BMI/M)}^{L}-1}{L \times S}$$

The symbols represent the following curves: L = skewness, M = median, and S = coefficient of variation [50, 51].

### Statistical analysis

In the presented study, data from one participant was not qualified for statistical analysis due to missing data in the Feelings and Attitudes Toward Body Scale questionnaire. In case of missing data in EAT 26 Questionnaire among 4,1% of participants, with regard to scoring procedure, interpolation was used to estimate up to one missing value for each subscale using the median subscale item value (rounding up to a whole number).

First, quantitative variables (body weight, height, BMI, body satisfaction, and training background) were presented as means and standard deviations. Qualitative variables (sport type and level of competition) were presented as numbers and percentage distributions. The Shapiro–Wilk test was used to examine whether the qualitative variables were normally distributed. Eating disorder risk was taken as the dependent variable, and the independent variables were body satisfaction, sport type, BMI, training background, and level of competition.

To assess the differences between the groups with eating disorder risk (ED+) and without eating disorder risk (ED-), the non-parametric Mann–Whitney U test (Z) was used due to the lack of a normal distribution in at least one of the compared groups. Differences between qualitative variables between the ED + and ED- groups depending on the results from the expected count tables were investigated using the V-square test (V^2^) and the Chi-square test (Chi^2^_NW_). In the case of significant differences between variables with more than two subcategories (BMI and sport type), further calculations with the two-sample test of proportions were performed.

To evaluate the relationship between two quantitative variables, a non-parametric Spearman’s rank correlation coefficient (r_s_) was used due to the absence of normal distribution in at least one of the variables. Differences between more than two subcategories of variables were analyzed using the non-parametric Kruskal–Wallis one-way analysis of variance (ANOVA) test.

In the next step, the assumptions for further analyzes were checked. The study met the conditions of dichotomy of the dependent variable (risk of eating disorders versus no risk of eating disorders), lack of significant extreme variables, independence of observation, homoscedasticity and linearity (p > .5). The collinearity condition was met under the assumption VIF < 2.5 [52]. The highest VIF value for variables in which collinearity was tested was equal to 1.2.

Finally, for all associations statistically significant between independent variables (body satisfaction, sport type, and level of competition) among athletes with and without eating disorder risk, univariate logistic regression analyses were performed (likelihood-ratio Chi-square test [LR], odds ratio [OR], and 95% confidence interval [CI]) to investigate the relationships between the occurrence of eating disorder risk (dependent variable, “yes” classification) and the independent variables. The threshold for statistical significance was set at *p <* .05. The results showed that the test power analysis for body satisfaction was 0.94, while for sport type was equal to 0.98. All calculations were made using Statistica data analysis software version 13.0 [53].

## Results

Table 2 presents a statistical description of the study group, including all the examined variables. In the total sample, 9.5% of athletes presented an eating disorder risk, made up of 14.6% of the adolescent athletes and 6.9% of the adult athletes.

Significant differences between athletes with and without eating disorder risk were reported only among the adolescents. In this age group, the number of underweight, normal weight, and overweight athletes varied significantly between the groups with and without eating disorder risk (*p =* .0060). Post hoc tests indicated that the percentage of overweight adolescent athletes with eating disorder risk was significantly higher than those without eating disorder risk (*p =* .0007). There were no subjects in the underweight category, which excluded the use of BMI in further analyses due to the lack of data to calculate the odds ratio. Regarding body satisfaction, adolescent athletes with eating disorder risk reported significantly lower body satisfaction than participants without eating disorder risk (*p =* .0032).

Among adolescents, the occurrence of eating disorder risk varied depending on the sport type (*p ≤* .0001 for all sport types). Post hoc tests showed that there were significantly fewer athletes without eating disorder risk in lean aesthetic sports than with eating disorder risk (*p* = .0106). In lean non-aesthetic sports, the number of athletes with eating disorder risk was significantly higher than the number of athletes without eating disorder risk (*p* < .0001). There were no statistically significant differences between the number of athletes with and without eating disorder risk in ball sports.

In the group of adolescent athletes, significant differences in eating disorder risk were observed between different levels of competition (*p = .*0330). Eating disorder risk was associated with a national level of competition. Training background (*p* = .2676), body weight (*p = .*4951), and height (*p =* .2510) did not differ significantly between athletes with and without eating disorder risk.

In contrast to the adolescents, among the adult athletes, there were no significant differences between individuals with and without eating disorder risk regarding any of the studied variables. Therefore, only the results from the adolescent athletes were subjected to further statistical analysis.

**Table 2:** Comparison of the characteristics of adolescent and adult athletes with and without eating disorder risk

Characteristic	Adolescent athletes (*n* = 82)			Adult athletes (*n* = 159)			
	ED+	ED-		ED+	ED-		
	*n* = 12	*n* = 70	*p*-value	*n* = 11	*n* = 148		*p*-value
	***M*** **±** ***SD*** **or % (** ***n*** **)**			***M*** **±** ***SD*** **or % (** ***n*** **)**			
Body weight (kg)	57.2 ± 10.39	53.5 ± 14.68	.4951^a^	63.5 ± 8.63		61.7 ± 8.83	.2205^a^
Height (cm)	161.9 ± 9.17	165.3 ± 13.59	.2510^a^	168.7 ± 1.97		170.0 ± 7.49	.9621^a^
BMI status							
Underweight	0	14 (10)	.0060^b^	0		19 (13)	.3322^b^
Normal weight	66 (8)	82 (57)		91 (10)		86 (128)	
Overweight	33 (4)^*^	4 (3)		9 (1)		5 (7)	
Body satisfaction (pt)	11.4 ± 6.42^*^	17.4 ± 4.40	.0032^a^	15.3 ± 6.42		18.2 ± 4.51	.1845^a^
Sport type							
Lean aesthetic	8 (1)^*^	44 (31)	≤ .0001^b^	0		16 (24)	.1428^b^
Lean non-aesthetic	59 (7)^*^	4 (3)		36 (4)		26 (38)	
Ball	33 (4)	52 (36)		64 (7)		58 (86)	
Level of competition							
National	58 (7)^*^	27 (19)	.0330^c^	55 (6)		31 (46)	.2050^c^
International	42 (5)	73 (51)		45 (5)		69 (102)	
Training background (years)	6.63 ± 2.50	5.63 ± 2.27	.2676^a^	8.36 ± 4.32		10.66 ± 4.67	.1099^a^

*Note*: ^a^Mann–Whitney U test; ^b^Chi^2^_NW_ test; ^c^V-square test; ^*^p < .05, significantly different from ED-.

BMI = body mass index; ED + = with eating disorder risk; ED- = without eating disorder risk; *M* – mean; *SD* = standard deviation; % (*n*) = percent (number); pt = point

Table 3 summarizes the relationships between the examined variables in adolescent athletes. There were no subjects in the underweight category (Table 1), which excluded the use of BMI in further analyses due to a lack of data to calculate the odds ratio.

**Table 3:** Relationships between the dependent variable (eating disorder risk) and the independent variables among adolescent athletes

Variables	1	2	3	4
1. Eating disorder risk	-			
2. Body satisfaction	0.0032^*^	-		
	(Z = 2.95)^a^			
3. Sport type	< 0.0001^*^	0.0141^*^	-	
	(Chi^2^_NW_ = 21.15)^b^	(H = 8.52)^e^		
4. Level of competition	0.0704	0.2498	0.1273	-
	(Chi^2^ = 3.27)^c^	(Z = -1.15)^a^	(Chi^2^_NW_ = 4.12)^b^	
5. Training background	0.2676	0.7582	0.0010^*^	0.0421^*^
	(Z = -1.11)^a^	(r_s_ = -0.03)^d^	(H = 13.77)^e^	(Z = -2.03)^a^

*Note*: ^a^Mann–Whitney U test; ^b^Chi^2^_NW_ test; ^c^V-square test; ^d^Spearman’s rank correlation coefficient; ^e^Kruskal–Wallis one-way ANOVA by ranks; ^*^p < .05, significantly different from ED+

Eating disorder risk was significantly related to body satisfaction and sport type (Table 3). Athletes with eating disorder risk had lower body satisfaction (*p = .*0032). The results also indicated a relationship between the sport type and body satisfaction (*p = .*0141). Training background differed between types of sport (*p* = .0010). Moreover, training background was significantly associated with the level of competition (*p* = .0421).

In the next step, univariate logistic regression analyses were performed with eating disorder risk as the dependent variable. The independent variables’ associations with eating disorder risk among adolescent athletes are presented in Table 4. Athletes practicing lean non-aesthetic sports were 11.5 times more likely to develop eating disorder risk than athletes practicing aesthetic or ball sports (OR = 11.50, 95% CI: 3.58–37.09). The risk of eating disorders was also associated with a lower level of body satisfaction (OR = 0.80, 95% CI: 0.70–0.92).

**Table 4:** Results of a univariate logistic regression **analyses** of eating disorder risk among adolescent athletes

Variable	Rate	OR	95% CI		*p*-value
			LL	UL	
**Model I**					
Body satisfaction	-0.22	0.80	0.70	0.92	0.002
**Model II**					
Sport type					
Lean aesthetic	-1.84	0.16	0.04	0.67	0.013
Lean non-aesthetic	2.44	11.50	3.58	37.09	*<* 0.001
Ball	-0.60	0.55	0.19	1.58	0.264

*Note*: CI = confidence interval, LL = lower limit, OR = odds ratio, UL = upper limit

To determine the quality of the relationships pertaining to the risk of occurrence of eating disorders, based on the variables selected for the model, an area under the ROC curve (AUC) was calculated and was found to be 0.902 (*±* 0.05), indicating that the model was characterized by good discrimination.

## Discussion

The aim of the study was to evaluate the relations between eating disorder risk among adolescent and adult female athletes and body satisfaction, sport type, BMI, level of competition, and training background. Significant relationships were found only in adolescent athletes. The results of a univariate logistic regression analyses demonstrated that adolescent athletes practicing lean non-aesthetic sports were at greater risk of eating disorders. However, while the OR was quite large for sport type, it should be noted that there was a large variation in the CI, likely due to the small number of participants practicing lean non-aesthetic sports. This probably had an impact on the results obtained. A weak relationship was also found between eating disorder risk and a low level of body satisfaction.

Many authors have highlighted that athletes participating in sports that are focused on appearance and low body weight are at greater risk of developing eating disorders [1, 3, 4, 16, 54]. In the present study, athletes practicing lean non-aesthetic sports were 11 times more likely to have eating disorder risk. Among the 12 adolescent athletes with eating disorder risk, seven practiced lean non-aesthetic sports, four were involved in ball sports, and one person participated in an aesthetic sport. This is an interesting and unexpected result; however, it should not be overestimated due to the small number of athletes in the eating disorder risk group. Nevertheless, some authors have reported similar inconsistencies in larger samples than that used in the present study. For example, Haase [31] investigated a sample of 137 female athletes participating in ball sports and lean non-aesthetic sports (aerobics and diving). The author found that lean non-aesthetic sport athletes exhibited a higher level of dieting and bulimic behavior than ball sport athletes and concluded that sport type predicted disordered eating.

In the present study, lean non-aesthetic sports included endurance sports, such as athletics and swimming, and weight-dependent sports, such as karate and taekwondo [7]. This inclusion might explain the observed relation between eating disorder risk and participation in lean non-aesthetic sports. Karate and taekwondo are sports in which athletes often fast and use pathogenic methods of body weight control [19]. Dietary and training restrictions implemented by athletes participating in sports with weight categories often contribute to the development of eating disorders [29, 30, 55, 56]. Furthermore, endurance sports, such as running, track and field, and swimming, are considered high-intensity sports and have been found to be related to eating disorder development among athletes [57, 58]. For endurance and track and field athletes, a higher body fat percentage can negatively affect performance [59]. In these sports, high-level athletes are often over-trained, present fatigue symptoms, and believe that thinness increases their performance [5]. They mostly achieve the desired body type through pathological eating behaviors that often develop into eating disorders [1, 60]. In addition, these athletes expose their bodies because they often wear very revealing outfits when training and competing.

In the case of swimming, it is not only artistic swimmers who are exposed to the pressures of weight reduction. Among the coaches and athletes involved in distance swimming, there is a perception that lower body weight and body fat improve swim times [61, 62]. Thus, athletes often use unhealthy eating techniques that can lead to eating disorders [5, 63]. Moreover, swimsuits and athletic costumes expose the body. This exposure to outside scrutiny causes additional pressure on athletes to conform to societal expectations regarding body shape, which may increase athletes’ pursuit of the desired physical appearance through inadequate nutritional practices.

The relationship between eating disorders and sport type among athletes requires further research. Some authors have suggested that the relationship may be reversed, and individuals with eating disorders may engage in sports that strengthen and manifest their disease [1, 64, 65]. This is a controversial statement that contradicts the results of other authors, which clearly indicated the relationship between eating disorders and sport type [3, 4, 16]. The present study revealed the presence of eating disorder risk among adolescent athletes in lean non-aesthetic sports and indicated the need to extend and refine the research on all types of sports.

In the present study, eating disorder risk among adolescent athletes was related to lower body satisfaction, which confirms the results of other studies [17, 22, 60, 66]. Krentz and Warschburger [22] found that, among variables such as gender, age, BMI, and body dissatisfaction, the latter was the strongest predictor of developing an eating disorder among elite athletes. In the present study, adolescent athletes with eating disorder risk had significantly lower body satisfaction than those without. This observation is in line with the literature, which suggests that body dissatisfaction and eating disorders are linked among competitive athletes [4, 16, 25, 67]. Researchers have argued that athletes with a negative body image are individuals who are afraid to gain weight, so they often diet and develop eating disorders [61, 68]. The sports environment provides many reasons for athletes to be concerned about their body weight. Athletes’ body stereotypes have their origins in the beliefs among the sports community that thin is beautiful and promotes sports performance and that, for males, a muscular body is masculine [60]. Therefore, the athletic environment creates expectations that athletes in certain sports should possess a characteristic body size or shape. For example, female gymnasts should be “tiny” or football players very muscular [17]. These stereotypes promote an unrealistic body image, and conforming to these expectations necessitates unhealthy eating behaviors among athletes. Therefore, consistent with the social comparison theory, some athletes have a low level of body satisfaction and suffer from eating disorders due to their attempts to achieve a specific athletic physique [25]. Moreover, female athletes in some sports (throwing, swimming, and wrestling) might have lower body satisfaction because their bodies deviate from the social ideals of a thin body [23, 69]. Kosteli et al. [23] stated that the body image of athletes varies depending on the sport type: athletes participating in aesthetic sports conform more to the cultural expectations regarding the female body, which may result in higher body satisfaction, compared to athletes practicing lean non-aesthetic and non-lean sports. A different approach to the problem was proposed by de Bruin et al. [3, 4, 16], who distinguished between athletic body image related to sport and daily body image. The authors reported a different relationship between eating disorders and daily or athletic body image among elite athletes. This highlights a future line of research.

When examining the importance of BMI in the occurrence of eating disorder risk, based on the first stages of analysis in the present study, it can be concluded that there were more overweight individuals with eating disorder risk than without. This is in line with a reported positive correlation between BMI and eating disorders among athletes [69, 70]. The obtained results suggest two possibilities: either overweight athletes use disordered eating to reduce their BMI, or they become overweight because of eating disorder behaviors, which is characteristic, for example, in binge eating [69]. However, BMI could not be included in the final model due to the lack of subjects in the underweight category and available data to calculate the odds ratio. The results suggest the secondary role of BMI in relation to body satisfaction and sport type in the occurrence of eating disorder risk among adolescent athletes. It is also possible that the relationship between eating disorder risk and BMI is linked to another variable which was not tested in this study. However, the results of the present study are in line with those of Thiemann et al. [71], who found that, when other variables were included, body dissatisfaction was a stronger predictor of eating disorder risk and counteracted the BMI effect.

In the present study, 14.6% of adolescent athletes presented eating disorder risk; in the adult group, this percentage was 6.9%. Significant differences between athletes with and without eating disorder risk occurred only in the adolescent group. This is in line with other studies which suggested that younger athletes were more prone to eating disorders than older athletes [72]. Krentz and Warschburger [22] assessed 96 elite athletes in aesthetic sports with a mean age of 14 years and found that age was one of the determinants of eating disorder prevalence. According to Nordin et al. [73], eating disorders commonly develop between 14 and 21 years of age. De Bruin et al. [16] found that 47% of elite and 51% of national-level adolescent athletes reported pathogenic methods of weight control. Other research reported that, among 53 highly trained elite athletes between 11 and 27 years old, the athletes with eating disorders were significantly younger [4]. These data indicate the need for separate studies on adolescent and adult groups.

The results of the present study showed that among adolescents, 58% of the athletes competing at a national level had eating disorder risk and 27% did not. This difference was statistically significant. Among adult athletes, no differences were reported between the groups with and without eating disorder risk. It has been determined that one of the triggers of eating disorders related to sports is early involvement in sports [74]. Younger athletes may be more vulnerable to developing eating disorders as they are more exposed to sport and sociocultural pressures regarding the ideal athletic body, performance, and competition [1]. They have also less training experience than older athletes. Both groups are influenced by various factors, some of which are related to the development of eating disorders, while others have a protective effect. Among high-level athletes, there is intense pressure to win and a high volume of training [11, 73]; however, they are often supported by qualified staff and their own experience [40]. National-level athletes often do not have this support system and the skills to cope with the stress and challenges associated with competitive sports, which can lead to unhealthy eating behaviors [74].

This study had some limitations. First, body satisfaction and eating disorder risk were assessed using a self-assessment method; therefore, the participants’ subjective interpretation of the questions may have influenced the results. Second, the EAT-26 is a screening tool biased toward the “thin body” ideal and may not detect the risk of some types of eating disorders. The results should therefore be interpreted with caution. Finally, the limited number of participants practicing lean non-aesthetic sports and the associated wide CI could have distorted the results.

The main strength of this paper was the inclusion of individual and sport environment factors in the analysis of eating disorder risk. Moreover, the study used a detailed division of sports into lean aesthetic, lean non-aesthetic, and ball sports. This allowed for the expansion of the knowledge about the risk of eating disorders among athletes participating in various types of sport. Another strength of this study was the separate analysis of adolescent and adult athletes.

## Conclusions

The results of the present study indicated that there are different relationships between eating disorder risk and individual (body satisfaction and body mass index) and sport environment factors (sport type, level of competition [national and international], and training background) among adolescent and adult athletes. Adolescent athletes are more at risk than adults of developing eating disorders. The main finding of this study is that eating disorder risk is the highest among athletes practicing lean non-aesthetic sports. Moreover, the results suggested that athletes with eating disorder risk are characterized by lower body satisfaction. Coaches and specialists should foster a positive body image among athletes and pay special attention to the eating behaviors of adolescent athletes, particularly those who practice lean non-aesthetic sports.

## Data Availability

The datasets generated or analyzed in this study are not publicly available because of ethical considerations but are available from the corresponding author on request.

## Abbreviations

BMI: Body mass index
CI: Confidence interval
EAT: Eating Attitudes Test
ED+: Athletes with eating disorder risk
ED-: Athletes without eating disorder risk
L: Skewness
LL: Lower limit
M: Mean value
OR: Odds ratio
S: Coefficient of variation
SD: Standard deviation
UL: Upper limit
% (n): Percent (number)
